# Frailty syndrome and associated factors among patients with hypertension: A cross‐sectional study in Kumasi, Ghana

**DOI:** 10.1002/hsr2.1664

**Published:** 2023-10-25

**Authors:** Samuel A. Sakyi, Phyllis Tawiah, Ebenezer Senu, Ransford O. Ampofo, Anthony K. Enimil, Benjamin Amoani, Enoch O. Anto, Stephen Opoku, Alfred Effah, Elizabeth Abban, Joseph Frimpong, Emmaunel Frimpong, Lydia Oppong Bannor, Afia A. Kwayie, Emmanuel Naturinda, Eugene A. Ansah, Bright T. Baidoo, Kini E. Kodzo, Nana K. Ayisi‐Boateng

**Affiliations:** ^1^ Department of Molecular Medicine Kwame Nkrumah University of Science and Technology Kumasi Ghana; ^2^ Department of Medicine, School of Medicine and Dentistry Kwame Nkrumah University of Science and Technology Kumasi Ghana; ^3^ Department of Medical Diagnostics, Faculty of Allied Health Sciences Kwame Nkrumah University of Science and Technology Kumasi Ghana; ^4^ Pediatric Infectious Disease Unit, Child Health Directorate Komfo Anokye Teaching Hospital Kumasi Ghana; ^5^ Department of Biomedical Science University of Cape Coast Cape Coast Ghana; ^6^ Department of Medical Laboratory Technology Garden City University College Kumasi Ghana

**Keywords:** antihypertensives, frailty syndrome, high blood pressure, hypertension, medication adherence

## Abstract

**Background and Aim:**

Frailty is a condition marked by accumulation of biological deficits and dysfunctions that come with aging and it is correlated with high morbidity and mortality in patients with cardiovascular diseases, particularly hypertension. Hypertension continues to be a leading cause of cardiovascular diseases and premature death globally. However, there is dearth of literature in sub‐Saharan Africa on frailty syndrome among hypertensives on medication. This study evaluated frailty syndrome and its associated factors among Ghanaian hypertensives.

**Methods:**

This cross‐sectional study recruited 303 patients with hypertension from the University Hospital, Kwame Nkrumah University of Science and Technology (KNUST), Kumasi, Ghana. Data on sociodemographic, lifestyle and clinical factors were collected using a well‐structured questionnaire. Medication adherence was measured using Adherence in Chronic Disease Scale, and frailty was assessed by Tilburg Frailty Indicator. Statistical analyses were performed using SPSS Version 26.0 and GraphPad prism 8.0. *p‐*value of < 0.05 and 95% confidence interval (CI) were considered statistically significant.

**Results:**

The prevalence of frailty was 59.7%. The proportion of high, medium and low medication adherence was 23.4%, 64.4% and 12.2%, respectively. Being ≥ 70years (adjusted odds ratio [aOR]: 8.33, 95% CI [3.72–18.67], *p* < 0.0001), unmarried (aOR: 2.59, 95% CI [1.37–4.89], *p* = 0.0030), having confirmed hypertension complications (aOR: 3.21, 95% CI [1.36–7.53], *p* = 0.0080), medium (aOR: 1.99, 95% CI [1.05–3.82], *p* = 0.0360) and low antihypertensive drug adherence (aOR: 27.69, 95% CI [7.05–108.69], *p* < 0.0001) were independent predictors of increased odds of developing frailty syndrome.

**Conclusion:**

Approximately 6 out of 10 Ghanaian adult patients with hypertension experience frailty syndrome. Hypertension complications, older age, being unmarried, and low antihypertensive drug adherence increased the chances of developing frailty syndrome. These should be considered in intervention programmes to prevent frailty among patients with hypertension.

## 1 INTRODUCTION

Hypertension continues to be a leading cause of cardiovascular disease and premature death globally. It is one of the most common conditions among the general population, contributing negatively to worldwide health and financial issues.^1^ It is estimated that over 1.28 billion persons worldwide ranges from the ages of 30–79 have hypertension, with approximately 67% residing in low‐ and middle‐income countries.^2^ In Ghana, hypertension is one of the top reasons of hospitalizations and fatalities. It was Ghana's third most significant cause of hospitalization and death in 2017, accounting for 4.7% of all hospital admissions and 15.3% of all deaths.^3^ In Ashanti region of Ghana, hypertension affects 37.4% of the population.^4^

Patients' adherence to antihypertensives is crucial to achieve their effects of reduction in stroke incidence, prevention of heart failure exacerbation and low risk of mortality.^5^, ^6^ About 55% of individuals with hypertension do not follow therapy recommendations, and this has been attributed to low socioeconomic level, comorbidities, age, physical limitations, or frailty syndrome.^7^

Frailty is a syndrome marked by the aggregation of biological deficiencies and dysfunctions resulting from ageing and disrupts the organism's homeostatic balance.^8^ It is a significant issue, mostly in older adults, with negative consequences for illness outcomes and treatment adherence.^9^ Frailty raises the likelihood of undesirable health outcomes such as mortality, disability, poor quality of life, hospitalization and institutionalization by making people more vulnerable to stress.^10^ The prevalence of this condition among community‐dwelling older individuals is significant, ranging from 8% to 16%.^11^ Frailty is associated with higher morbidity and mortality in patients suffering from cardiovascular disease.^12^, ^13^ It has been proposed that recognizing frailty can aid clinicians in determining procedure risks, evaluating prognosis, and guiding management.^14^

Frailty syndrome in older individuals has received a lot of attention lately. It is estimated 15%–20% of people over 60% and 30% of patients over 80 are affected by frailty syndrome.^15^ With the life expectancy of Ghanaians at 64 years,^16^ the prevalence of frailty may be higher at even younger ages in the general Ghanaian population. It is advised that, better knowledge of frailty in cardiac care, especially in older adults should be enhanced.^17^ In all, 25%–50% of patients with a cardiac disease suffer from frailty syndrome.^15^ Some of these include patients with hypertension. Despite the increasing prevalence of frailty, its associated factors, including medication adherence among patients with hypertension have not been explored in sub‐Saharan Africa. For the first time, we determined the prevalence and predictors of frailty syndrome in a sample of Ghanaian patients with hypertension.

## 2 MATERIALS AND METHODS

### 2.1 Study design

This study was a cross‐sectional study design. The study sought to determine the prevalence and factors associated with frailty syndrome among patients with hypertension from Janury 2022 to September 2022.

### 2.2 Study site

**Figure 1:**
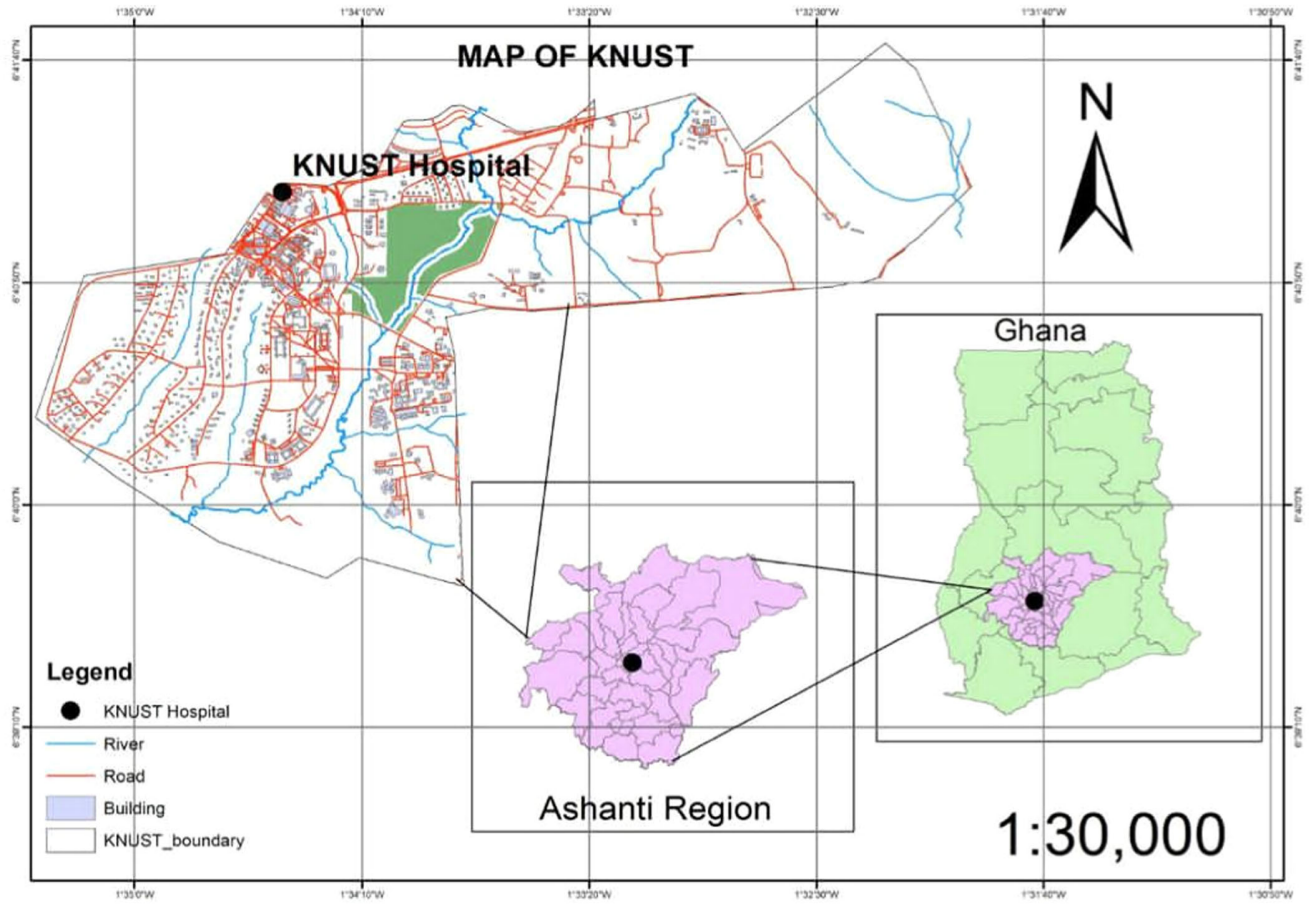
Map of University Hospital, Kwame Nkrumah University of Science and Technology (KNUST), Kumasi, Ghana.

The study was conducted at the University Hospital, Kwame Nkrumah University of Science and Technology (KNUST) in Kumasi, Ghana. Kumasi is the regional capital of the Ashanti Region of Ghana, with an estimated population of 200,000 (Figure 1). The hospital offers services in general medical care as well as specialist services. The geographical location of the 135‐bed hospital, and the road network of Kumasi make the hospital accessible. As such, referrals are received from other centers, making our study population quite representative.

### 2.3 Study population

The study population included adult patients (40 years and above) with clinically diagnosed hypertension in the outpatient department (OPD) at the University Hospital, KNUST, Kumasi.

### 2.4 Sample size

The sample size was estimated using the Cochran formula;

$n=\frac{Z^{2}\;p\left(1-p\right)}{e^{2}}$; where: *Z* is the standard normal variate at a confidence interval (CI) of 95% = 1.96, *p* is the estimated prevalence of hypertension (16.6%) in Ashanti region,^18^ *e* is the margin of error (0.05).

$$n\;(\text{Minimum number of participants})=\frac{1.96^{2}(0.166)\left(1-0.166\right)}{0.05^{2}}=213.$$

The minimum number of participants required was 213. However, we enrolled 303 participants into the study to increase the statistical power.

### 2.5 Inclusion and exclusion criteria

The study enrolled clinically diangosed hypertensives aged 40 years and above, who were on at least one antihypertension medication. Patients below the age of 40 years, patients with no history of hypertension, and patients who had not been treated with any antihypertensive drug or with cognitive impairments were excluded from the study.

### 2.6 Ethical consideration

Ethical approval was sought from the Committee on Human Research, Publication and Ethics, School of Medicine and Dentistry, Kwame Nkrumah University of Science and Technology (CHRPE/SMS/KNUST/CHRPE/AP/421/22). We sought the voluntary participation of individuals and obtained written informed consent from each participant.

### 2.7 Data collection

We used a well‐structured questionnaire to collect sociodemographic, lifestyle, and clinical data from participants. In addition, standardized instruments, which included Adherence in Chronic Disease Scale (ACDS)^19^ and Tilburg Frailty indicator (TFI), were used to measure adherence and frailty, respectively, among study participants. Before the commencement of the study, these questionnaires were piloted among hypertensives at Kumasi South Government Hospital, Kumasi, and reliability was determined using Cronbach's *α*.

### 2.8 Measurement of medication adherence using ACDS

To assess study participants' compliance with medication adherence, we employed the ACDS. The underlying assumption of ACDS is that in pharmacology, only great adherence is indicative of an effective therapeutic plan. There were seven questions on the scale, and each had five options. Questions 1–5 dealt with behaviors that directly influence adherence, whereas questions 6–7 dealt with circumstances and viewpoints that do the same. The ACDS score ranges from 0 to 28 points. Higher results mean higher adherence. Thus, above 26 points indicated good adherence, between 21 and 26 points indicated medium adherence, and below 21 points indicated low adherence.^19^

### 2.9 Measurement of frailty syndrome using the TFI

The TFI was created by Gobbens et al.^20^ TFI is divided into two components. The participant's sociodemographic data are covered in part one, and 15 self‐reported questions are separated into three categories: social, psychological, and physical. Eight questions in the physical domain are scored from 0 to 8 points and pertain to physical health, unexplained weight loss, difficulties walking, balance, hearing and vision issues, hand strength, and physical exhaustion. The psychological domain consists of four items (0–4 points each) that address cognition, depressive symptoms, anxiety, and coping. The social domain consists of three questions (0–3 points) about interacting with others, receiving social support, and living alone. Eleven of the TFI's second part's questions have only two possible answers: yes or no, whereas the other questions have three (yes, no, and occasionally). “Yes” or “Sometimes” responses receive one point apiece, while “no” responses receive zero points. The total score might be between 0 and 15: The fragility syndrome increases with score. Frailty is diagnosed at a total TFI score ≥ 5.^20^ The TFI has been proven to be reliable and valid for assessing frailty.

### 2.10 Instrument validity and reliability

The questionnaire was designed based on validated questions from previous studies. Before the study commenced, the questionnaire was pre‐tested in different hypertensive populations at the Suntreso Hospital in Kumasi, and all misleading and ambiguous questions were corrected. Moreover, the internal consistency for the reliability of the ACDS and TFI questionnaire was computed using Cronbach's *α*.

### 2.11 Statistical analysis

Data obtained from participants were coded into Microsoft Excel 2019 and analyzed using Statistical Package for Social Sciences (SPSS) Version 26.0 and GraphPad prism version 8.0 (GraphPad software; www.graphpad.com). Categorical variables were presented as frequencies and percentages. Univariate and multivariate logistic regression prediction models were employed to predict the association between study variables and frailty syndrome using frailty syndrome as the dependent variable. A *p*‐value of < 0.05 and a 95% CI were considered statistically significant.

## 3 RESULTS

### 3.1 Sociodemographic and clinical characteristics of study participants

Out of the 303 participants, the majority (35.3%) were 70 years and above, and most (57.8%) of them were females. Over two‐third (71.6%) of the study participants were married. Considering educational level, 35.3% had primary education, with 50.5% having secondary to tertiary education (Table 1).

**Table 1:** Sociodemographic characteristics of study participants.

Variable	Frequency (*n* = 303)	Percentage (%)
Age group (Years)		
40–49	57	18.8
50–59	71	23.4
60–69	67	22.1
70 and above	108	35.3
Gender		
Male	128	42.2
Female	175	57.8
Marital status		
Married	217	71.6
Unmarried	86	28.4
Education level		
No formal education	43	14.2
Primary	107	35.3
Secondary	91	30
Tertiary	62	20.5
Occupation		
Unemployed	89	29.4
Informal	143	47.2
Formal	41	13.5
Retired	30	9.9
Family history of hypertension		
No	164	54.1
Yes	139	45.9

### 3.2 Clinical and lifestyle characteristics of study participants

The majority (61.1%) of the study participants were taking other medications besides the antihypertensives. Over two‐thirds (78.2%) of the study participants had no confirmed hypertension complications. Most of the participants (61.4%) answered yes to the presence of other medical conditions. A good majority of the study participants had diabetes in addition to hypertension (59.1%). They formed 96.2% (179 of 186) of those with other known medical. Approximately half (50.8%) of the study participants visited the hospital every 3 months, with 63.0% not engaging in physical exercise. For those who engaged in physical exercise, 59.8% exercised occasionally, followed by weekly (27.7%), whilst few (12.5%) engaged in daily exercises. Regarding the timing of meals and snack intake,63.7% of the participants did not take snacks in between meals, and 64.4% took meals late at night (Table 2).

**Table 2:** Clinical and lifestyle characteristics of study participants.

Variable	Frequency (*n* = 303)	Percentage (%)
Any drug apart from HPT drug		
No	118	38.9
Yes	185	61.1
Confirmed HPT complication		
No	237	78.2
Yes	66	21.8
Any other medical conditions		
No	117	38.6
Yes	186	61.4
Diabetes		
No	124	40.9
Yes	179	59.1
Other conditions		
No	288	95
Yes	15	5
Group		
Hypertension alone	124	40.9
Hypertension and Diabetes	179	59.1
Hospital visits		
Monthly	33	10.9
2 months	116	38.3
3 months	154	50.8
Exercise		
No	191	63
Yes	112	37
Frequency of exercise		
Daily	14	12.5
Weekly	31	27.7
Occasionally	67	59.8
Snacks in between meals		
No	193	63.7
Yes	40	13.2
Sometimes	70	23.1
Meals late at night		
No	195	64.4
Yes	13	4.3
Sometimes	95	31.4

Abbreviation: HPT, hypertension.

### 3.3 Prevalence of antihypertensive drug adherence and frailty among study participants

**Figure 2:**
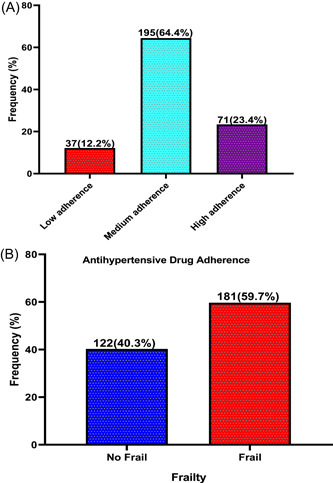
Prevalence of antihypertensive drug adherence (A) and frailty (B) among study participants.

The majority (64.4%) of the study participants had medium adherence to their antihypertensive drugs, followed by high adherence (23.4%), and 12.2% had low adherence (Figure 2A). On the other hand, more than half (59.7%) of the participants had frailty syndrome (Figure 2B).

### 3.4 Sociodemographic predictors of frailty syndrome

In a univariate logistic regression model, compared to participants within 40–49 years, those who were 70 years and above (crude odds ratio [cOR]: 10.05, 95% CI [4.59–21.98], *p* < 0.0001) had higher odds of experiencing frailty. Also, those unmarried (cOR: 2.93, 95% CI [1.66–5.16], *p* < 0.0001) and participants who had a family history of hypertension (cOR: 1.96, 95% CI (1.22–3.14), *p* = 0.0050) had higher odds of frailty. However, gender, educational level, occupation, ethnicity, and religion, all had *p* > 0.05, and these were excluded from the multivariate logistic regression model analyses.

After adjusting for possible cofounders, age, and gender in a multivariate logistic regression model, being 70 years and above (aOR: 8.33, 95% CI [3.72–18.67], *p* < 0.0001) and being unmarried (aOR: 2.59, 95% CI [1.37–4.89], *p* = 0.0030) were independently associated with increased odds of developing frailty syndrome (Table 3).

**Table 3:** Sociodemographic predictors of frailty syndrome.

Variable	Frail (*n* = 181)	cOR (95%CI)	*p*‐Value	aOR (95%CI)	*p*‐Value
Age group (years)					
40–49	24 (13.3)	1.00	‐	1.00	‐
50–59	27 (14.9)	0.84 (0.41–1.72)	0.64	0.75 (0.36–1.58)	0.448
60–69	35 (19.3)	1.50 (0.74–3.06)	0.261	1.43 (0.68–3.02)	0.348
70 and above	95 (52.5)	10.05 (4.59–21.98)	**<0.0001**	8.33 (3.72–18.67)	**<0.0001**
Gender					
Male	80 (44.2)	1.22 (0.77–1.95)	0.402	1.26 (0.73–2.16)	0.413
Female	101 (55.8)	1.00	‐	1.00	‐
Marital status					
Married	115 (63.5)	1.00	‐	1.00	‐
Unmarried	66 (36.5)	2.93 (1.66–5.16)	**<0.0001**	2.59 (1.37–4.89)	**0.003**
Education level					
No formal education	27 (14.9)	1.00	‐	‐	‐
Primary	61 (33.7)	0.79 (0.38–1.63)	0.516	‐	‐
Secondary	56 (30.9)	0.95 (0.45–2.00)	0.889	‐	‐
Tertiary	37 (20.4)	0.88 (0.39–1.95)	0.748	‐	‐
Occupation					
Unemployed	66 (36.5)	1.23 (0.49–3.07)	0.657	‐	‐
Informal	70 (38.7)	0.41 (0.18–1.10)	0.056	‐	‐
Formal	24 (13.3)	0.61 (0.22–1.64)	0.324	‐	‐
Retired	21 (11.6)	1.00	‐	‐	‐
Family history of hypertension					
No	86 (47.5)	1.00	‐	1.00	‐
Yes	95 (52.5)	1.96 (1.22–3.14)	**0.005**	1.63 (0.95–2.79)	**0.075**

*Note*: Bolded values are significant, *p* = < 0.05.

Abbreviations: aOR, adjusted odds ratio; cOR, crude odds ratio.

### 3.5 Clinical and lifestyle predictors of frailty syndrome

In a univariate logistic regression model, taking any medication apart from the antihypertensives (cOR: 3.30, 95% CI (2.03–5.35), *p* < 0.0001), having confirmed hypertension complications (cOR: 5.77, 95% CI (2.73–12.19), *p* < 0.0001), having other medical conditions (cOR: 3.00, 95% CI [1.86–4.86], *p* < 0.0001), history of diabetes (cOR: 3.17, 95% CI [1.96–5.12], *p* < 0.0001) and having both hypertension and diabetes (cOR: 3.17, 95% CI [1.96–5.12], *p* < 0.0001) were associated with increased odds of developing frailty syndrome. However, engaging in exercises (cOR: 0.31, 95% CI [0.19–0.50], *p* < 0.0001), taking snacks in between meals (cOR: 0.48, 95% CI [0.24–0.95], *p* = 0.0350) and not regularly taking meals late at night (cOR: 0.42, 95% CI [0.26–0.70], *p* = 0.0010) were respectively associated with 31%, 48%, and 42% lower chances of developing frailty syndrome.

After adjusting for possible confounders in a multivariate logistic regression model, having confirmed hypertension complications (aOR: 3.21, 95% CI [1.36–7.53], *p* = 0.0080) was independently increased the odds of developing frailty syndrome (Table 4).

**Table 4:** Clinical and lifestyle predictors of frailty syndrome.

Variable	Frail (*n* = 181)	cOR (95%CI)	*p‐*Value	aOR (95%CI)	*p*‐Value
Any drug apart from HPT drug					
No	50 (27.6)	1.00	‐	1.00	‐
Yes	131 (72.4)	3.30 (2.03–5.35)	**<0.0001**	1.42 (0.60–3.35)	0.425
Confirmed HPT complication					
No	124 (68.5)	1.00	‐	1.00	‐
Yes	57 (31.5)	5.77 (2.73–12.19)	**<0.0001**	3.21 (1.36–7.53)	**0.008**
Any other medical conditions					
No	51 (28.2)	1.00	‐	1.00	‐
Yes	130 (71.8)	3.00 (1.86–4.86)	**<0.0001**	0.74 (0.17–3.18)	0.688
Diabetes					
No	54 (29.8)	1.00	‐	1.00	‐
Yes	127 (70.2)	3.17 (1.96–5.12)	**<0.0001**	1.17 (0.29–4.68)	0.825
Other conditions apart from diabetes					
No	171 (94.5)	1.00	‐	‐	‐
Yes	10 (5.5)	1.37 (0.46–4.11)	0.576	‐	‐
Group					
HPT	54 (29.8)	1.00	‐	1.00	‐
HPT‐DM	127 (70.2)	3.17 (1.96–5.12)	**<0.0001**	1.17 (0.29–4.68)	0.825
Hospital regularity					
Monthly	25 (13.8)	1.00	‐	‐	‐
2 months	65 (35.9)	0.41 (0.17–1.28)	0.065	‐	‐
3 months	91 (50.3)	0.46 (0.20–1.09)	0.078	‐	‐
Exercise					
No	134 (74)	1.00	‐	1.00	‐
Yes	47 (26.0)	0.31 (0.19–0.50)	**<0.0001**	0.67 (0.35–1.27)	0.223
How often do you exercise					
Daily	7 (14.9)	1.23 (0.39–3.91)	0.721	‐	‐
Weekly	10 (21.3)	0.59 (0.24–1.44)	0.243	‐	‐
Occasionally	30 (63.8)	1.00	‐	‐	‐
Snacks in between meals					
No	122 (67.4)	1.00	‐	1.00	‐
Yes	18 (9.9)	0.48 (0.24–0.95)	**0.035**	1.52 (0.59–3.91)	0.384
Sometimes	41 (22.7)	0.82 (0.47–1.44)	0.495	2.04 (1.00–4.17)	0.050
Meals late at night					
No	131 (72.4)	1.00	‐	1.00	‐
Yes	6 (3.3)	0.42 (0.14–1.30)	0.131	1.79 (0.42–7.63)	0.429
Sometimes	44 (24.3)	0.42 (0.26–0.70)	**0.001**	1.18 (0.59–2.34)	0.639

*Note*: Bolded values mean statistically significant, *p* = <0 .05.

Abbreviations: aOR, adjusted odds ratio; cOR, crude odds ratio; HPT, hypertension; HPT‐DM, hypertension and diabetes mellitus.

### 3.6 Medication adherence as predictors of frailty syndrome

**Figure 3:**
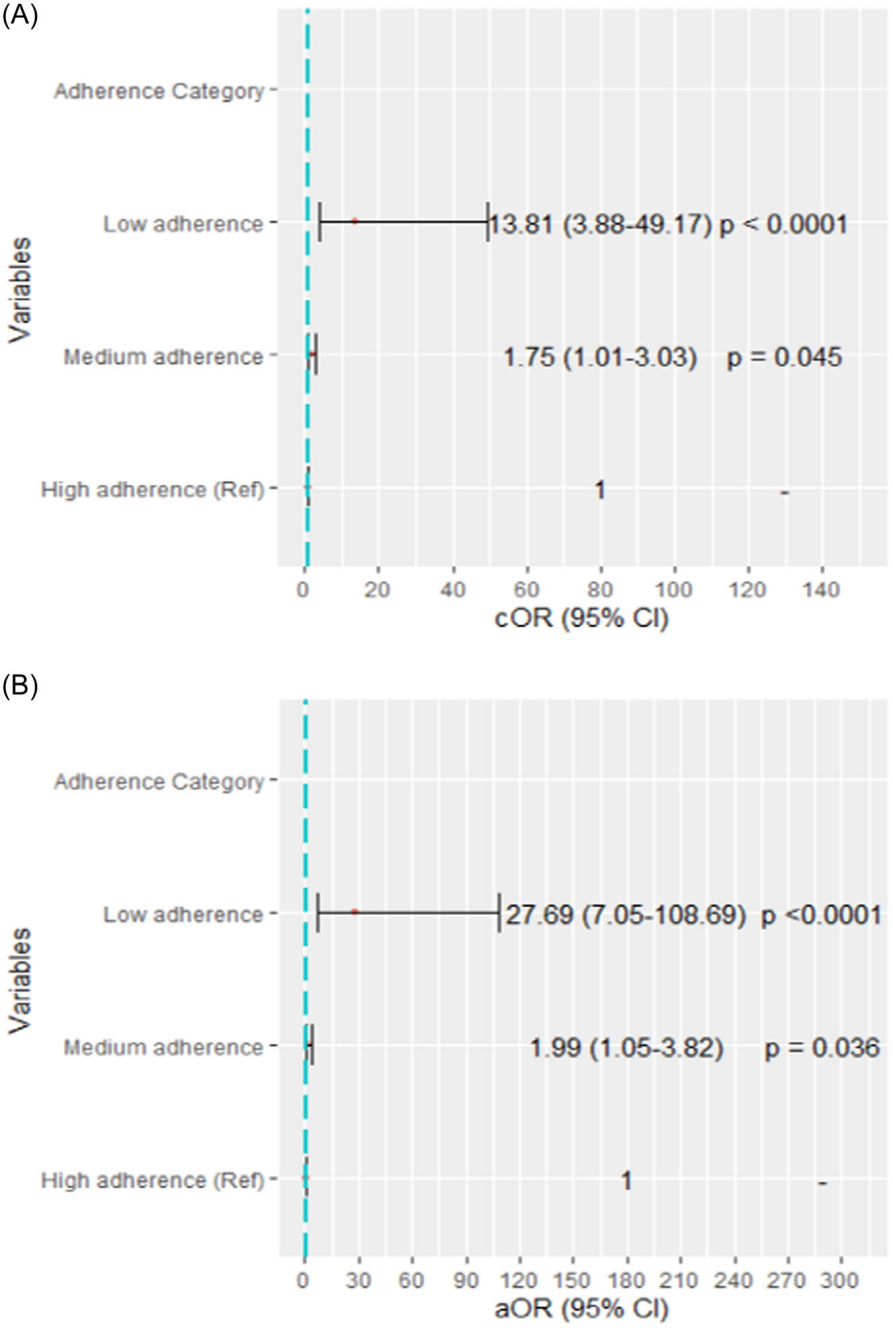
Medication adherence as a predictor of frailty syndrome; univariate (A) and multivariate (B) logistic regression analyses predicting frailty syndrome among hypertensives, reference = 1.00. aOR, adjusted odds ratio; cOR, crude odds ratio.

In a univariate logistic regression model, participants with medium antihypertensive adherence (cOR: 1.75, 95% CI [1.01–3.03], *p* = 0.0450) and low antihypertensive adherence (cOR: 13.81, 95% CI [3.88–49.17], *p* < 0.0001) were associated with higher odds of having frailty syndrome compared to high adherence (Figure 3A).

After adjusting for possible confounders, in a multivariate logistic model, medium (aOR: 1.99, 95% CI [1.05–3.82], *p* = 0.0360) and low antihypertensive drug adherence (aOR: 27.69, 95% CI [7.05–108.69], *p* < 0.0001) independently increased odds of developing frailty syndrome compared to high adherence (Figure 3B).

## 4 DISCUSSION

Hypertension continues to be a leading cause of cardiovascular disease and premature death globally. Despite the increasing prevalence of frailty, its associated factors, including medication adherence among patients with hypertension, have not been explored in Sub‐Saharan Africa. In this study, the prevalence of frailty among the study participants was 59.7%. Previous studies have reported frailty prevalence among community dwelling older adults in Ghana as 37.9%.^21^ Our finding is much higher than the proportion of frailty found by Aprahamain et al.^22^ (14.8%). In their study, though conducted in an outpatient center like ours, the inclusion criterion was open to all older adults presenting to the center and not focused on individuals having hypertension as a baseline. The significant difference, therefore, may likely be due to the underlying hypertension in all our participants and the high prevalence of combined hypertension and diabetes mellitus. Likewise, Ma et al.^23^ reported a frailty proportion of 13.8% among older adults in China, which is much lower compared to what was obtained in this current study. Again, their study looked at community‐dwelling older adults with individuals diagnosed with hypertension and those undiagnosed, but had measured systolic blood pressures of over 140 and diastolic blood pressures over 90 during the study. We believe these differences are partly related to the varying socio‐genetic factors but, more importantly, the fact that we focused on a population who were all hypertensives seeking medical care for that with over 50% having concomitant diabetes mellitus.

We found being 70 years and above, being unmarried, having confirmed hypertension complications and medium and low antihypertensive drug adherence were independently associated with increased odds of developing frailty syndrome. Female participants were more likely to be frail than their male counterparts and age was also found to be an independent predictor of frailty syndrome. These findings are consistent with results from Gobbens and Assen,^24^ who reported age as a determinant of frailty and reported females to be more affected than males. In another study, Fattori et al.,^25^ who examined a group of Brazilian patients, also found that the prevalence of frailty syndrome was higher in females than in males. The present study further confirms increasing age, female sex, and presence of chronic medical diseases, especially in older adults, as independent risk factors of frailty. Furthermore, it reinforces the significant impact of the latter.

Despite the findings above, women have documented higher life expectancies than men in most countries.^26^, ^27^ This inconsistency in the likelihood of frailty and mortality in females is likely due to the effect of riskier lifestyles in men, such as smoking, alcohol use, that are associated with higher cardiovascular risk.^28^ Moreira and Lourenco.^29^ reported marital status was associated with the development of frailty. In their study, individuals who were unmarried were at increased risk of developing frailty syndrome. This is consistent with our study in which being unmarried was an independent predictor of frailty syndrome. The positive association could be attributed to the psychological, emotional and physical support provided by married couples that has been found to limit depression and the likelihood of frailty. Similarly, a study by Monin et al.^30^ revealed that, an individual's frailty is predicted by the spouse's or partner's relationship effects. Furthermore, our study found the presence of hypertension complications as an independent predictor of frailty syndrome. In this study, the proportion of high adherence among hypertension patients was 23.4%. This was lower compared to similar local studies, one at Korle‐Bu Teaching Hospital, Ghana (47.7%),^31^ and a multicenter study in Ghana and Nigeria (33.3%). On the other hand, Sarkodie et al.^32^ reported a much lower adherence to antihypertensive medication (7.0%), at the Komfo Anokye Teaching Hospital, Ghana. These differences in medication adherence may be due to variations in the methodologies used in measuring medication adherence aside the socioeconomic factors and beliefs identified in these studies. This study revealed low medication adherence as an independent predictor of frailty syndrome. The finding is in line with a study by Jankowska et al.,^7^ who found that adherence was lower in people with frailty syndrome.

This is the first study to report frailty syndrome among patients with hypertension in Ghana, Although the prevalence of frailty was high because of the study settings and population, the determinants are consistent with that of studies from other populations. The findings may be limited. Our analysis did not include other chronic conditions besides hypertension and diabetes. Secondly, this study did not include drug interactions and adverse effects in the case of individuals on other medications aside from antihypertensive medication. We therefore recommend that future studies should include these factors to assess more potential risk factors of frailty syndrome among patients with hypertension.

## 5 CONCLUSION

We found frailty to be present in approximately 6 out of 10 Ghanaian adult patients with hypertension, and that the presence of hypertension complications, advancing age, not being married, and low antihypertensive drug adherence increase the chances of developing frailty syndrome. Therefore, we recommend interventions aimed at preventing frailty among patients with hypertension to include measures that limit these factors.

## AUTHOR CONTRIBUTIONS

**Samuel Asamoah Sakyi**: Conceptualization; data curation; formal analysis; investigation; methodology; supervision; writing—original draft; writing—review & editing. **Phyllis Tawiah**: Conceptualization; data curation; investigation; methodology; resources; writing—original draft; writing—review & editing. **Ebenezer Senu**: Conceptualization; data curation; formal analysis; investigation; methodology; validation; writing—original draft; writing—review & editing. **Ransford Osei Ampofo**: Conceptualization; data curation; formal analysis; investigation; methodology; resources; validation; writing—original draft; writing—review & editing. **Anthony Kwame Enimil**: Conceptualization; data curation; formal analysis; investigation; methodology; resources; supervision; validation; writing—original draft; writing—review & editing. **Benjamin Amoani**: Conceptualization; data curation; formal analysis; investigation; methodology; resources; writing—original draft; writing—review & editing. **Enoch Odame Anto**: Conceptualization; data curation; formal analysis; investigation; methodology; validation; writing—original draft; writing—review & editing. **Stephen Opoku**: Conceptualization; data curation; formal analysis; investigation; methodology; writing—original draft; writing—review & editing. **Alfred Effah**: Conceptualization; data curation; formal analysis; investigation; methodology; writing—original draft; writing—review & editing. **Elizabeth Abban**: Conceptualization; data curation; formal analysis; investigation; methodology; writing—original draft. **Joseph Frimpong**: Data curation; formal analysis; investigation; methodology; writing—original draft; writing—review & editing. **Emmaunel Frimpong**: Conceptualization; data curation; formal analysis; methodology; writing—original draft; writing—review & editing. **Lydia Oppong Bannor**: Conceptualization; data curation; methodology; writing—original draft; writing—review & editing. **Afia Agyapomaa Kwayie**: Conceptualization; data curation; investigation; methodology; writing—original draft; writing—review & editing. **Emmanuel Naturinda**: Conceptualization; data curation; methodology; writing—original draft; writing—review & editing. **Eugene Arele Ansah**: Conceptualization; data curation; methodology; writing—original draft; writing—review & editing. **Bright Takyi Baidoo**: Conceptualization; data curation; methodology; writing—original draft; writing—review & editing. **Kini Evans Kodzo**: Conceptualization; data curation; methodology; writing—original draft; writing—review & editing. **Nana Kwame Ayisi‐Boateng**: Conceptualization; data curation; formal analysis; investigation; methodology; resources; supervision; writing—original draft; writing—review & editing.

## CONFLICT OF INTEREST STATEMENT

The authors declare no conflict of interest.

## TRANSPARENCY STATEMENT

The lead author Ebenezer Senu affirms that this manuscript is an honest, accurate, and transparent account of the study being reported; that no important aspects of the study have been omitted; and that any discrepancies from the study as planned (and, if relevant, registered) have been explained.

## Data Availability

All data generated or analyzed during this study are included in this article and can be requested from the corresponding author.
